# Blood Brain Barrier: A Challenge for Effectual Therapy of Brain Tumors

**DOI:** 10.1155/2015/320941

**Published:** 2015-03-19

**Authors:** Arijit Bhowmik, Rajni Khan, Mrinal Kanti Ghosh

**Affiliations:** Signal Transduction in Cancer and Stem Cells Laboratory, Division of Cancer Biology and Inflammatory Disorder, Council of Scientific and Industrial Research-Indian Institute of Chemical Biology (CSIR-IICB), 4 Raja S.C. Mullick Road, Jadavpur, Kolkata 700 032, India

## Abstract

Brain tumors are one of the most formidable diseases of mankind. They have only a fair to poor prognosis and high relapse rate. One of the major causes of extreme difficulty in brain tumor treatment is the presence of blood brain barrier (BBB). BBB comprises different molecular components and transport systems, which in turn create efflux machinery or hindrance for the entry of several drugs in brain. Thus, along with the conventional techniques, successful modification of drug delivery and novel therapeutic strategies are needed to overcome this obstacle for treatment of brain tumors. In this review, we have elucidated some critical insights into the composition and function of BBB and along with it we have discussed the effective methods for delivery of drugs to the brain and therapeutic strategies overcoming the barrier.

## 1. Introduction

Brain is the most delicate organ of human body. Several diseases like encephalitis, neurological disorders, multiple sclerosis, stroke, and tumor induce deterioration of brain function. The development of new therapeutic approaches for these diseases is a difficult challenge, and there is no effective treatment for almost all the brain diseases. In most of the cases, the major cause of the failure in the development of drugs to treat brain diseases is the presence of BBB. Out of the several brain disorders, brain tumors commonly have poor prognosis, which varies according to the type and grade of the tumor. Due to the presence of BBB, drug delivery to brain tumors has long been a problematic issue. Some group of researchers like Vick et al. and Donelli et al. mentioned BBB as a controversial problem for brain tumor chemotherapy [1, 2]. They indicated that BBB is not the only factor responsible for impeding the success of brain tumor chemotherapy, but later, studies revealed the involvement of BBB in drug restriction to different brain neoplasias [3–6].

Brain tumors can be classified into two major classes, namely, primary brain tumors that start in the brain and secondary brain tumors that are generated by the cancer cells that migrated from tumors developed in other parts of the body. Primary brain tumors can arise from different type of brain cells or even from the membranes around the brain (meninges), nerves, or glands. The most common type of primary tumors in the brain is glioma, which arises from the glial tissue of the brain. Gliomas comprise several types, namely, astrocytoma, oligodendroglioma, and ependymomas. Astrocytomas are further classified as grade I (pilocytic), grade II (fibrillary), grade III (anaplastic), and grade IV (glioblastoma multiforme or GBM). BBB is poorly developed in these types of brain tumors causing an increased vascular permeability [7].

It has been shown earlier that leaky interendothelial tight junction is present in human glioma [8] due to the fact that poorly differentiated neoplastic astrocytes do not release factors essential for BBB function [9–11]. This tight junction opening causes increased chances of cerebral edema occurrence [12]. It is also observed that BBB stability in lower grade gliomas is better than that in GBM. As the degree of BBB disruption differs from the malignancy of the tumor, treatment of low grade brain tumors is still a challenging task, because of the presence of almost intact BBB. On the contrary, recent studies have suggested that although the BBB may be disrupted at or near the core of the high grade brain tumors, most certainly it seems to be intact near the growing edge of the tumor where the invasive tumor cells may reside. The presence of the intact BBB in such regions of the tumors can considerably impede drug delivery to these regions [13–15]. On the other hand, lack of BBB has been observed in other primary brain tumors like meningiomas, schwannomas, or pineocytomas [16–18]. Disrupted BBB also exists in metastatic secondary brain tumors, but the disruption is negligible in smaller aggregates of metastatic tumor cells. Therefore, the drug delivery to these micrometastatic regions is not optimum; consequently, the tumor keeps growing and ultimately reaches to clinically significant size. Thus, along with the existing therapeutic modalities, new approaches of therapy are needed to combat against the BBB of different brain tumors (see Table 1).

## 2. BBB

BBB protects neural tissues in the brain and works as a diffusion barrier that impedes the influx of toxins and other compounds from blood to the brain. BBB was discovered in 1880s. It took almost 70 years to successfully prove the existence of BBB by electron microscopic cytochemical studies [19, 20]. Later, in 1981 Stewart and Wiley explained the initial understanding about the uniqueness of BBB tight junction and its physiology [21].

**Figure 1:**
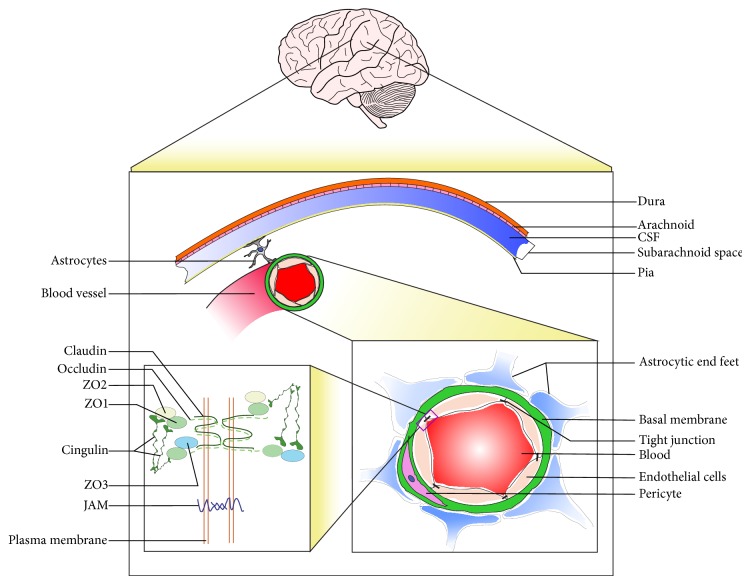
A pictorial representation of the BBB and its tight junction structure. The figure shows an irrigated blood vessel in the brain which forms the BBB. The BBB is constituted by endothelial cells with tight junctions, surrounded by pericytes and astrocytic end-feet. The tight junction is further established by the interaction of proteins like claudins, occludin, junction adhesion molecules, and cytoplasmic accessory proteins (ZO1, ZO2, and ZO3) of adjacent endothelial cells. The details of each component of the BBB are mentioned in the text of this review.

Molecular character of BBB shows the presence of two types of cellular junctions, the intercellular adherens junction and the paracellular tight junction. The functional integrity of BBB is maintained by adherens junction that is composed of vascular endothelium (VE), cadherin, actinin, and catenin [22]. But the major functionality of BBB is maintained by tight junctions, as they are primarily responsible for permeability through BBB [23, 24]. The BBB in adult is comprised of a complex cellular network. The main components of this system are brain endothelial cells, highly specialized basal membrane, a plenty of pericytes embedded in the basal membrane, and astrocytic end-feet (see Figure 1).

*Brain Endothelial Cells.* These cells are required for proper barrier formation and interaction with the adjacent cells. They are also known as brain microvascular endothelial cells (BMECs). The BMECs differ from the endothelial cells present in the other organs in the following ways: (i) paracellular movement of molecules is prevented by continuous tight junctions present between brain endothelial cells, (ii) BMECs have few cytoplasmic vesicles and more mitochondria, and (iii) detectable transendothelial path like intracellular vesicular transport is not present in BMECs [22, 25]. Complex intercellular tight junctions restrict the passive diffusion of molecules into the brain and therefore the blood vessels showing extremely high transendothelial electrical resistance (TEER)* in vivo* [26]. BMECs are also endowed with the ability to shuttle essential nutrients and metabolites across the BBB, which include molecules, like efflux transporters (p-glycoprotein). These transporters contribute to the BBB properties by efflux of small lipophilic molecules that are able to diffuse into BMECs, back to the blood stream. 

*Basal Membrane.* It consists of type IV collagen, fibronectin, and laminin that completely covers the capillary endothelial cell layers. Pericytes are embedded in this membrane and surrounded by astrocytic end-feet. The potential function of this membrane is to restrict the movement of the solutes [27, 28]. 

*Pericytes.* The contractile cells which are wrapped around the endothelial cells are called pericytes. These cells play an essential role in the formation of BBB in several ways such as by regulating the expressions of BBB-specific genes in endothelial cells by inducing polarization of astrocytic end-feet surrounding CNS blood vessels, and also they inhibit CNS immune cells from damaging the proper formation of BBB. Besides, these cells also help in reduction of the expression of molecules that increase vascular permeability [29].

*Astrocytic End-Feet.* It is assumed earlier that the astrocytic end-feet encircling endothelial cells do not play substantial role in maintenance of BBB [30]. But recent study by Nuriya et al., 2013, indicated the heterogeneity of diffusion patterns around astrocytic end-feet [31]. They proved the existence of some astrocytic end-feet which can form tight networks that are able to block free diffusion of molecules across them. The types of blood vessels and morphological differences in the gliovascular interface like the space between the endothelial cells and astrocytic end-feet determine the heterogeneity of diffusion patterns. Thus, these networks cover the blood vessels tightly which suggests the potential functional roles of astrocytic end-feet [32].

### 2.1. Molecular Composition of BBB

The tight junction of BBB mainly consists of three main integral membrane proteins, namely, occludin, claudin, and junction adhesion molecules. Other than that, cytoplasmic accessory proteins like zonula occludens (ZO 1, ZO 2, ZO 3, etc.), cingulin, and others are also present in BBB (see Figure 1).

*Occludin.* It is the first transmembrane protein of the tight junction to be discovered. Occludin was first identified in 1993 by immunogold freeze fracture microscopy in chicken [33] and then in mammals [34]. It is formed by four transmembrane domains: a long carboxy-terminal cytoplasmic domain, a short amino-terminal cytoplasmic domain, and two extracellular loops. The ZO proteins are directly associated with cytoplasmic domain of occludin. Phosphorylation of specific Ser/Thr/Tyr residues of occludin regulates its interaction with ZO proteins which in turn plays a regulatory role in tight junction formation [35]. 

*Claudins.* These are a multigene family of at least 24 members. They form tight junctions through homophilic “claudin-claudin” interactions mediated by their extracellular loops [36]. Carboxy terminal of claudins binds to the cytoplasmic proteins including ZO family members [37]. Occludins and claudins can also assemble into heteropolymers to form intramembranous strands. It has been proposed that these strands contain fluctuating channels, which allow the selective diffusion of ions and hydrophilic molecules [38]. Claudins-1, -3, -5, and -12 have been shown to participate in the formation of tight junctions between BMECs [9, 10, 39, 40]. Each claudin regulates the diffusion of a group of molecules of specific size. 

*Junction Adhesion Molecules (JAM).* These proteins belong to the immunoglobulin superfamily. Three JAM-related proteins, JAM-A, JAM-B, and JAM-C, have been investigated in rodent brain sections. In human, it is observed that JAM-A and JAM-C are expressed in the tight junctions of BBB but not JAM-B [41]. JAM-B can be found in seminiferous epithelial cells [42]. All JAM proteins comprise a single transmembrane domain; the extracellular portion has two immunoglobulin like loops. They regulate the formation of tight junctions during the acquisition of cell polarity [43]. 

*Cytoplasmic Accessory Proteins*. Cytoplasmic proteins like zonula occludens proteins (ZO 1, ZO 2, and ZO 3), cingulin, 7H6, and several others are also involved in tight junction formation. Zonula occludens are proteins belonging to the family of membrane associated guanylate kinase (MAGUK) [44]. They provide the cytoskeletal anchorage for the transmembrane tight junction and control spatial distribution of claudins [24]. Cingulins are actomyosin-associated proteins with large globular N-terminal “head” domain, coiled-coil “rod” domain, and small globular C-terminal “tail.” Cingulin helps in BBB formation by interacting with ZO proteins and junction adhesion molecules.

### 2.2. Transporters of BBB

Endogenous compounds and drugs may cross BBB by different mechanisms such as passive diffusion, carrier-mediated transport (like GLUT1 mediated transport), endocytosis, and active transport [45–52]. Participation of various transport proteins is there in most of these transport systems. These different transport proteins of brain mediate the uptake and extrusion of various metabolites and compounds. The efflux and influx transporter systems of BBB comprise transporters like ATP-binding cassette (ABC) transporters and solute carrier (SLC) transporters.

#### 2.2.1. ABC Transporters

ABC (ATP-binding cassette) transporters are ATP-driven drug efflux pumps present in the BBB which include P-glycoprotein, breast cancer resistance protein, and members of the multidrug resistance related proteins [53]. These proteins form a key characteristic of the BBB by localizing at the luminal side of brain capillaries. They collectively impede brain uptake of a large variety of lipophilic molecules, xenobiotics, potentially toxic metabolites, and drugs. ABC transporters show broad substrate specificity and have been characterized by one or two cytoplasmically located nucleotide binding domains acting as a catalytic domain for nucleotide hydrolysis. There are 48 genes encoding ABC transporter superfamily of proteins, which are subdivided into 7 distinct subfamilies (ABCA to ABCG) [54]. All ABC transporters have three highly conserved motifs known as Walker A, Walker B motifs and the ABC signature C motif (i.e., ALSGGQ) [55]. It has been suggested that this domain may be involved in substrate recognition and ATP hydrolysis [56].

*(1) P-glycoprotein (P-gp).* It is a 170-kDa efflux transporter discovered in Chinese hamster ovary cells [57]. P-gp is encoded by multidrug resistant (MDR) genes [58]. Two MDR isoforms have been identified in human tissues, MDR-1 and MDR-2 [59, 60]. MDR1 encoded P-gp is a major efflux transporter of BBB, the expression of which is likely evolved to protect the brain from exposure to potentially neurotoxic xenobiotics. Thus, it is considered that P-gp has a key role in the maintenance of accurate homeostatic environment required for proper neuronal function [61]. The MDR1 gene product is 1280 amino acids in length and has two homologous halves; each consists of six transmembrane domains and ATP- binding site. On the first extracellular loop, two to four glycosylation sites are present [62]. In the brain, P-gp is localized to both the luminal and abluminal sides of BBB endothelium [63] and to the apical plasma membrane of choroid plexus epithelial cells [64]. Substrates of P-gp are usually nonpolar, weakly amphipathic compounds which significantly vary in molecular size. The different types of endogenous substrates of P-gp include cytokines, lipids, steroid hormones, and peptides [65]. P-gp has a vast endogenous and exogenous substrate profile that renders difficulty in drug delivery across the BBB.

*(2) Breast Cancer Resistance Protein (BCRP).* It was first identified in the MCF-7/AdrVp breast cancer cell line [66]. It is also known as a “half-transporter.” Its molecular weight is approximately 72 kDa and it is composed of 655 amino acids. It has six transmembrane domains and both the C- and N-terminus regions are located on the intracellular side of the plasma membrane [67].

Furthermore, the extracellular loops of the protein contain two to three sites for N-linked glycosylation. According to the earlier reports, the functional capabilities of the transporter and its cellular localization are not dependent on these glycosylation sites [67, 68]. It is also known that BCRP forms functional homo- or heterodimers to maintain the efflux activity [69]. BCRP is expressed at the luminal side of capillary endothelial cells, in astrocytes and microglia [70–72]. The substrate specificity of BCRP not only is limited to the physiological substrates, such as glutathione, steroid hormones, and folic acid [73], but also transports many structurally diverse therapeutic compounds. Significantly, the specificity of BCRP to the substrates overlaps with the substrate specificity of P-gp [74]. It is also known that high expression of these BCRP proteins causes significant resistance to different cancer chemotherapeutic drugs [75, 76].

*(3) Multidrug Resistance Proteins (MRPs).* It is well established that MRP family has 9 homologues, designated as MRP1–9, and these isoforms have overlapping substrate profiles. Out of these, expression of MRP1–6 has been observed in human brain [77], whereas multiple MRPs like MRP1, 4, and 5 have been detected in the human BBB [72, 78, 79]. Existence of other MRPs, namely, MRP2, 3, and 6 and along with these MRP1, 4, and 5, has also been noticed in other vertebrates like rat, cow, pig, and fish. But presence of them in human BBB is still questionable. Structural similarity can be observed in MRP1, 2, 3, and 6, as each of them possesses 3 transmembrane domains (TMD) designated as TMD0, TMD1, and TMD2, respectively. TMD1 and TMD2 contain 6 alpha helices, whereas TMD0 contains only 5 alpha helices [80, 81]. It is believed that TMDs are assembled in the plasma membrane pore through which the transport of substrates occurs [80]. On the contrary, MRP4 and MRP5 have structural similarity with P-gp that lack TMD0 [80, 82], but in all the MRP homologues, the conserved cytoplasmic linker (L0) portion is essential for transport function. MRP1, 4, and 5 are restricted to the luminal membrane of human brain capillary endothelial cells [81]. The localization of MRPs suggests that they play a crucial role in drug efflux transport through BBB.

#### 2.2.2. Solute Carrier (SLC) Transporters

**Figure 2:**
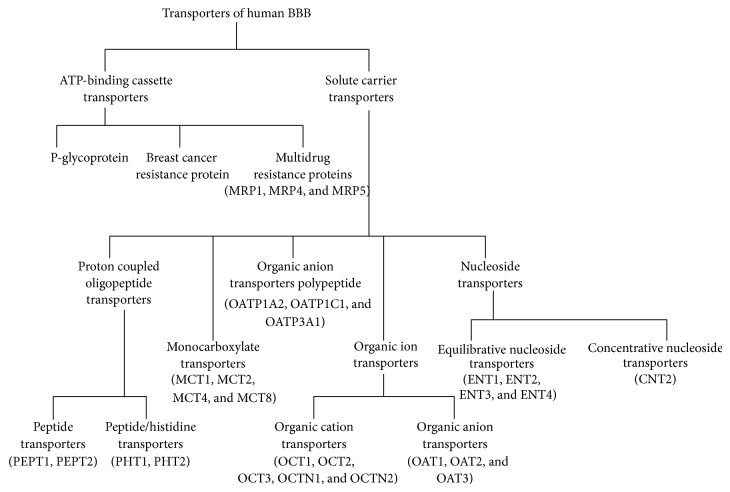
Schematic classification of transporters of human BBB. Two main classes of drug transporters are ATP-binding cassette (ABC) transporters and solute carrier transporters. Each of them is further classified into several other transporters mentioned in the flowchart. More information about each of the transporters is mentioned in the text.

SLC transporters belong to SLC superfamily which comprises 43 known subfamilies of SLC transporters (SLC1–SLC43). At the BBB, SLC15A1, SLC16, SLC21, SLC22, SLC28, and SLC29 are expressed [83]. The major SLC transporters include proton coupled oligopeptide transporters, monocarboxylate transporters, organic anion polypeptide transporters, organic ion (anion and cation) transporters, and nucleoside transporters (see Figure 2) [84, 85]. Most of these transporters of BBB regulate the transport of brain tumor drugs by hindering their entry into the tumor regions. Generally, these SLC transporters do not require ATP to translocate substrates across BBB; however, the electrochemical or concentration gradients of solute are essentially required for this type of transportation. 

*(1) Proton Coupled Oligopeptide Transporters (POT).* POT belongs to SLC15A family solute carrier transporters. Names of the subfamilies of POT are peptide transporters (PEPT) and peptide/histidine transporter (PHT). Peptide transporter-1 (PEPT1; SLC15A1) and peptide transporter-2 (PEPT2; SLC15A2) are the members of PEPT subfamily, whereas PHT comprises peptide/histidine transporter-1 (PHT1; SLC15A4) and peptide/histidine transporter-2 (PHT2; SLC15A3) [86, 87]. These oligopeptide transporters are able to transport small peptides across the BBB by an electrochemical proton gradient [88]. Structural similarity can be observed in POT family members due to the presence of 12 *α*-helical transmembrane domains with intracellularly located C- and N-terminal regions. Two to seven glycosylation sites exist in the extracellular loops, while intracellular loops have protein kinase A and C phosphorylation sites [86, 89]. Other than the above-mentioned peptide transporters, peptide uptake and distribution in brain are also determined by peptide transport system (PTS) expressed endogenously at the BBB endothelium [90]. In the BBB, seven transport systems have been found for transport of peptides, which includes PTS1–PTS7. PTSs, PTS2, PTS4, and PTS6, are bidirectional, whereas the rest are unidirectional. The unidirectional PTSs, PTS1 and PTS5, facilitate brain-to-blood peptide transport, whereas PTS3 and PTS7 are known for reverse process [90].

*(2) Monocarboxylate Transporters (MCTs).* Generally, the MCTs facilitate the rapid transport of monocarboxylates across the biological membranes. In brain, MCTs not only assist the transport of the monocarboxylates for uptake into the neurons but also mediate the transport of some drugs across the BBB [91]. These MCTs are members of solute carrier family 16 (SLC16). SLC16 has 14 members, out of which only six have been functionally characterized and those MCTs are MCT1–4, MCT8, and the T-type amino acid transporter-1 (TAT-1/MCT10) (326, 327). MCT1, MCT2, and MCT4 are the most important BBB transporters, whereas active MCT8 expression has also been detected in BBB [92–94]. The MCT1 protein is present in the membrane of the capillary endothelium and astrocytes, while MCT2 and MCT4 are found on neurons and astrocytes, respectively [95, 96]. 

*(3) Organic Anion Transporters Polypeptides (OATPs).* These membrane influx transporters are present in BBB to regulate cellular uptake of a number of endogenous compounds and clinically important drugs [97]. The human OATP comprises 11 members: OATP1A2, 1B1, 1B3, 1C1, 2A1, 2B1, 3A1, 4A1, 4C1, 5A1, and 6A1 [98–100], where OATP1A2 is the first discovered human member of the OATP family [101]. The OATP genes are classified within the SLCO (formerly SLC21A) family. Members of the same OATPs family share ~40% [99], whereas members of individual subfamilies possess ~60% amino acid sequence similarity. This group of transporters has broad substrate specificity. The OATP dependent transport of the substrates does not require ATP as energy source, yet it is conducted by electrochemical gradients that utilize an inorganic or organic solute as a driving force. The OATPs family members OATP1A2, 1C1, 2A1, 2B1, 3A1, and 4A1 are present in human brain [99]. OATP1A2 is the only human OATP isoform whose expression and function are widely established at BBB. The localization of OATP1A2 can be observed at both the luminal and abluminal membranes of human BBB endothelial cells [102]. The endogenous substrates of OATP1A2 are bilirubin, bromosulfophthalein, cholate, deltorphin-II, estradiol-17*β*-glucuronide, estrone-3-sulfate, glycocholate, hydroxyurea, PGE2, reverse-T3, taurocholate, taurochenodeoxycholate, tauroursodeoxycholate, T4, T3, and so forth [103], whereas a broad exogenous therapeutic substrate specificity can be noticed for this kind of OATPs. OATP1C1 and OATP3A1 are known to be present in both apical and basal sides of the brain endothelial cells and blood cerebrospinal fluid barrier, respectively, while the exact role of other OATPs is yet to be determined [104, 105]. 

*(4) Organic Ion Transporters.* These transporters can be classified into two specific types: (i) organic anion transporters (OATs) and (ii) organic cation transporters (OCTs). These transporters are the members of SLC transporter 22 superfamily (SLC22A) [83, 106]. 

*(i) Organic Anion Transporters (OATs).* The OAT family comprises OAT 1–6 and the renal specific transporter (RST) [107–111]. This classification is based on ATP-dependent energy requirements and involvement of Na^+^ ion. [112]. Movement of the organic anions across biological membranes is determined by these OATs. Various endogenous molecules like anionic metabolites of neurotransmitters, hormones, prostaglandins, and exogenous molecules such as different drugs are known to cross the biological membrane by these OATs [113]. The general structure of OATs comprises 12 membrane-spanning *α*-helices and several glycosylation and PKC sites, which can be found on extracellular loops connecting helices 6 and 7 [113]. In brain, OAT3 is the most highly expressed isoform. It is reported earlier that OAT3 is present in the abluminal (brain side) and brush-border membrane (CSF side) of brain capillary endothelial cells and choroid plexus epithelial cells, respectively [114, 115]. Other than this, OAT1, OAT2, and OAT4–6 are also expressed in brain [78, 105, 109, 114–117]. But the proper localization and function of these OATs are yet to be known. 

*(ii) Organic Cation Transporters (OCTs).* OCTs regulate the transport mechanisms to facilitate the passage of organic cations through biological membranes [118]. According to their transport capabilities, OCTs are categorized into two subgroups, namely, oligospecific organic cation transporters and polyspecific organic cation transporters. Apart from this, organic cation transporters can also be classified as chemical potential sensitive organic cation transporters (OCTs) and H^+^ gradient-dependent novel organic transporters (OCTNs). OCTs comprise OCT1–3, whereas OCTN transport system includes OCTN1 and OCTN2 [119]. Cellular influx and efflux of various cationic substrates are maintained by OCTs and OCTNs, respectively [120, 121]. All OCT family members generally contain 12 *α*-helical transmembrane domains with intracellular N- and C-termini. Furthermore, large extracellular loop between TMD1 and TMD2 and small intercellular loop connecting TMD6 and TMD7 are also present in OCT family members. In brain, OCT1–3 are localized to the basolateral membrane of BMECs and choroid plexus epithelial cells [122–124], and OCTN2 is reported to be localized to the luminal side of the BBB [125–127], whereas OCTN1 is reportedly absent in human CNS tissue [128]. Other than the transport of endogenous organic cations, OCT family members may also play crucial role in drug penetration through BBB [129].

*(5) Nucleoside Transporters.* The nucleosides play a major role as second messengers in many signal transduction pathways. Thus, their regulation of them is crucial for proper neuronal function [130]. The recycling pathways for nucleosides transportation into CNS tissue are needed, as brain cannot synthesize nucleosides* de novo*. Depending on the Na^+^ dependence nucleoside, the membrane transporters are again classified into two subcategories: equilibrative nucleoside transporters (ENTs) and concentrative nucleoside transporters (CNTs). ENTs are the members of the SLC29A transporter family and are Na^+^-independent, whereas CNTs are the members of the SLC28A transporter family and are Na^+^-dependent [131, 132]. In humans four isoforms of ENTs have been discovered, which are ENT1–4 [133–135]. All of them possess 11 *α*-helical transmembrane domains with intracellular N-terminus and extracellular C-terminus regions. Each and every isoform of ENTs also possesses a large cytoplasmic loop and an extracellular loop [136, 137]. ENT1, ENT2, and ENT4 are ubiquitously expressed in brain tissue and are localized to cellular membranes [138–140]. CNTs also have three isoforms: CNT1–3. They are integral membrane proteins with 13 transmembrane *α*-helices and a large extracellular C-terminal region, present in various regions of brain [85] and work as antiporters. CNT1 and CNT2 transport nucleosides into the cell in exchange for sodium ions, while CNT3 transports nucleosides in exchange for either sodium ions or protons [141]. But prominent expression of CNT2 protein has been observed at the luminal side of the BBB endothelium. Other than the endogenous nucleoside transporters, CNTs are also responsible for the cellular uptake of a number of nucleoside-derived drugs [85].

### 2.3. Aberrant Expression of BBB Components in Brain Tumors

BBB components claudins and occludins are either downregulated or not at all expressed in brain tumors. Loss of claudin-1 and downregulation of claudin-3 and claudin-5 expressions in high grade glioma are reported earlier [9, 142]. This variation of expression of claudins causes loosening of BBB tight junctions, but the involvement of claudins in the mechanism for the compromised tight junction function in BBB is not very clear. Claudin-1 proteins are known to regulate different signaling pathways, which in turn alter the expression and function of different cell-cell adhesion molecules [143]. It is also reported that claudin-5 regulates BBB permeability during the metastasis of brain tumors [144]. Loss of expression of another transmembrane protein occludin in microvessels is also observed in astrocytomas and metastatic adenocarcinomas. The probability of their contribution to endothelial tight junction opening is also very high [11]. High grade astrocytomas secrete vascular endothelial growth factor (VEGF), which downregulates the expression of occludins and increases endothelial cell permeability [145]. However, besides VEGF, cytokines and scatter factor or hepatocyte growth factor are also secreted by astrocytoma and other brain tumors. These factors are believed to be involved in the downregulation of tight junction molecules leading to its leakage [146, 147].

## 3. Drug Delivery Approaches and Current Advances in Brain Tumor Therapy

Most of the brain tumor drugs are ineffective due to their limited entry through BBB. Nowadays scientific communities are interested in providing solutions to this problem, and it is not surprising that most of the brain tumor patients could benefit from the improved drug delivery approaches. Few established approaches are intra-arterial drug delivery, intrathecal and intraventricular drug administration, intratumoral delivery, receptor-mediated transport, disruption of BBB, inhibition of drug efflux by BBB, and the use of intranasal drug delivery route.

The clinical trials of intra-arterial delivery in brain tumor drugs show minimal improvement in survival of brain tumor patients [148–154], but recently the neurosurgeons of New York Presbyterian Hospital/Weill Cornell Medical Center for the first time showed the successful intra-arterial delivery of monoclonal antibody like bevacizumab to the tumor region by means of transient blood brain barrier disruption [155]. In case of intrathecal drug administration, the drugs possess limited ability to enter the extracellular space of brain from the CSF [156–159]. The convection enhanced diffusion (CED) technique is used in transcranial brain drug delivery approaches to evade the BBB for forceful delivery of fluid into the brain and to increase the effective infiltration of drug into tumor region [160]. Application of microdialysis in neurooncology is also well established since it has been proposed as an efficient method of intratumoral drug delivery. This method employs the passive diffusion of a drug across the BBB [161, 162] and distributes drugs away from the dialysis catheter [163]. On the other hand, the receptor-mediated endocytosis and exocytosis facilitate the entry of the therapeutic compounds across the BBB of brain tumors. Receptor targeted monoclonal antibody-based drugs are delivered across the BBB by the help of receptor-mediated transport systems [164, 165]. Another traditional approach to solve the problem of drug delivery into the brain is BBB disruption. Osmotic disruption technique, bradykinin-analogue or alkylglycerol mediated disruption technique, MRI-guided focused ultrasound BBB disruption technique, and so forth are used to disrupt the BBB [166–169]. Though, bradykinin analogue mediated delivery of drug is abandoned due to its ineffectiveness when administered in combination with carboplatin. Recently, MRI-guided focused ultrasound BBB disruption technique is used to disrupt BBB for effective drug delivery [170].

P-glycoproteins (P-gp) of the ABC drug efflux transporters are present not only in low grade brain tumors but also in different malignant glioma cells [171]. Modulation of P-gp may cause effective delivery of drugs to the tumor niche. The poor* in vivo* efficacy of the first generation P-gp modulators (verapamil, cyclosporine A, tamoxifen, and several calmodulin antagonists) is due to their low binding affinities, which necessitated the use of high doses, resulting in intolerable toxicity [172]. The coadministration of the second-generation P-gp modulators (dexverapamil, dexniguldipine, valspodar (PSC 833), and biricodar (VX-710)) [173, 174] and chemotherapy agents in clinical trials has provided limited success; hence, third-generation P-gp modulators come in the scenario. These modulators include anthranilamide derivative tariquidar (XR9576), cyclopropyldibenzosuberane zosuquidar (LY335979), laniquidar (R101933), and elacridar (GF120918) [175–178]. Kemper et al. showed 5-fold increase in brain uptake of paclitaxel by combinatorial treatment with elacridar (GF120918) [178]. Other than P-gp inhibitors, MRP inhibitors (like sulfinpyrazone, probenecid, etc.) and BCRP inhibitors (fumitremorgin C and its analogues) are also reported as transporter inhibitors [172, 179]. Ongoing clinical trials with these new P-gp inhibitors should prove whether this approach will result in increased survival of brain tumor patients.

A promising drug delivery technique that can bypass the BBB is the usage of intranasal drug delivery route. This technique eliminates the risk of surgery and the nonspecific spillover effect of drug to normal tissue. Intranasal delivery provides successful drug targeting mechanism which utilizes the unique anatomic connections of olfactory and trigeminal nerves of nasal mucosa and the central nervous system [180, 181]. The drugs administered through this path reach the cerebrospinal fluid (CSF), spinal cord, and brain parenchyma very rapidly. This delivery system has been proven to be successful in delivering anticancer agents to the brain, like raltitrexed, 5-fluorouracil, GRN163, and methotrexate [182–185]. Further studies about intranasal therapeutic agents are needed and it could be a major candidate for clinical trials in brain tumor patients.

Current techniques and new approaches in drug delivery across the BBB can be classified as follows.

### 3.1. Modification of Existing Drugs

The ability of drug to cross the BBB depends on few factors like molecular size (should be less than 500 Da), charge (should have low hydrogen bonding capabilities), and lipophilicity (should have high lipophilicity) [186]. Thus, chemical modification of brain tumor drugs refers to the process of making an existing drug smaller in size, more perfectly charged, and more lipid soluble [187] (Table 2). Existing brain tumor drugs may also be modified to make analogue of the ligand to the particular receptor present in the BBB or the ligand or a peptide can be linked to a drug against the cellular receptors of BBB. The drug melphalan has been modified by using this approach where melphalan nitrogen mustard (mechlorethamine) was linked to phenylalanine [188]. Another approach of drug modification is the use of lipid carriers for efficient transport through BBB. One example of such modification is incorporation of small drugs in fatty acids like N-docosahexaenoic acid (DHA) [189, 190]. Drugs are also modified in such a way that they acquire increased capillary permeability, but after crossing the BBB they undergo an enzymatic reaction and return to their active state. This approach is also known as prodrug therapy [191, 192].

### 3.2. Nanosystem Based Delivery

Nanosystems are colloidal carriers that mainly consist of liposomes and polymeric nanoparticles while other systems, including solid lipid nanoparticles, polymeric micelles, and dendrimers, have also been studied recently. Sizes of these nanosystems vary within 1–1000 nm. These kinds of functionalized drug colloidal carriers can act as a vehicle to deliver antitumor drugs to brain tumor tissues. These nanosystems generally use passive diffusion mechanism as they rely on increased vascular permeability of brain tumor location, but usage of active chemically modified drugs with nanoparticles and receptor-mediated or adsorptive endocytosis processes of nanoparticle delivery have also been reported [219–221]. Conjugation of ligands targeting BBB on the surface of the nanosystem increases their specificity for brain tumors. One of the important features of these nanosystems is that they can circulate in the bloodstream for a prolonged time period. But the interaction of the nanosystems with the reticuloendothelial system (RES) causes its rapid removal from systemic circulation [222]. Therefore, to minimize the interactions of nanosystems with the RES, polyethylene glycol (PEG) coating or direct chemical linking of PEG to the particle surface is a widely accepted approach. These colloidal nanosystems comprise liposomes and nanoparticles, which have shown potential to target brain tumors as drug carriers. Furthermore, studies are going on for the development of novel transport-enhancing nanocarriers for brain tumor treatment.

#### 3.2.1. Liposomes

Liposome is a good carrier system for the delivery of therapeutic agents for brain tumors. They are easy to prepare, biocompatible, less toxic, and commercially available. Along with PEGylation, the liposomes can also be modified with monoclonal antibodies against transferrin receptors (OX-26), glial fibrillary acidic proteins (GFAP), or human insulin receptors [223]. Effective delivery of drugs like 5-fluorouracil (5-FU) and sodium borocaptate (Na210B12H11SH, BSH) to high grade brain tumors has been achieved by liposome mediated delivery [224, 225]. Modified liposomes like p-aminophenyl-*α*-D-mannopyranoside (MAN) and transferrin conjugated daunorubicin liposomes and* trans*-activating transcriptional peptide (TATp) modified liposomes have also been used* in vitro* and* in vivo *for targeting brain tumors [226, 227].

#### 3.2.2. Nanoparticles

Polymeric nanoparticles (NP) are colloidal particles which can be found in the form of nanocapsules or nanospheres. The drugs are dissolved, entrapped, encapsulated, adsorbed, or chemically linked to the surface of the NPs. The polymer structure and the drug trapping method determine the drug characteristics and its release kinetics from the nanoparticles [228]. One example of nanoparticle drug delivery approach is the usage of nanoparticles coated choline derivative that is reported to be transported across brain-derived endothelial cells by the cation transporter system [229]. Other remarkable systems are polysorbate-coated doxorubicin nanoparticles and doxorubicin-loaded folic acid-decorated nanoparticles, which cause effective penetration of drugs through BBB [230, 231]. Brain tumors can also be selectively targeted by bionanocapsules conjugated with anti-human EGFR antibody that recognizes EGFRvIII known to be overexpressed in high grade brain tumors like glioblastoma multiforme [232]. Those bionanocapsules may also contain virus, active proteins, vaccines, genes, or small interference RNA for targeted therapy of brain tumors. Solid lipid nanoparticles (SLNs), which are the dispersions of solid lipid stabilized with emulsifier or emulsifier/coemulsifier complex in water, are also known for delivering brain tumor drugs like camptothecin, doxorubicin, and paclitaxel to brain effectively [233]. Furthermore, gold nanoparticles and carbon nanoparticles (like carbon nanotubes, graphene, and carbon dots) are also able to deliver drugs (like doxorubicin) successfully [234–237]. Thus, nanoparticles may be considered as one of the most promising tools to deliver therapeutic drugs across the BBB to treat brain tumors [238].

Other nanosystems like polymeric micelles and dendrimers are also effective for targeted delivery of drugs to the tumors in the brain. Formation of polymeric micelles occurs spontaneously in aqueous solutions of amphiphilic block copolymers, whereas dendrimers are highly branched polymer molecules formed by a central core. These types of nanopreparations loaded with anticancer drugs should be considered as highly potential antitumor nanomedicines as they have the ability to cross the BBB by modulating BBB transporters like P-gp or glucose transporters [239–241].

### 3.3. Delivery Systems Used in Gene Therapy

Effective treatment of brain tumor can be obtained from intracerebral implantation of a therapeutic gene, inserted into a viral vector. It is a specifically targeted therapy where volume of the implantation is very low (<1 mm^3^). Thus, the expression of exogenous gene is highly localized. But gene reformulation may cause the generalised expression of exogenous gene in the total brain tumor niche. Few examples of carriers in this type of therapeutic systems are viral vectors like adenovirus, herpes simplex virus (HSV), and nonviral gene delivery system like cationic liposome-DNA complexes [242–244]. The O6-methylguanine-DNA methyltransferase (MGMT) upregulation in GBM makes it resistant to Temozolomide (TMZ), a well-known drug for glioma. Therefore, upregulation of wild-type (wt) p53 expression is needed which downmodulates MGMT. Since p53 therapy for GBM is not very efficient due to the presence of the blood brain barrier (BBB), a systemic nanodelivery platform (scL) for tumor-specific targeting (primary and metastatic) has been developed by Kim et al. It has been observed that the combination of scL-p53 and TMZ increased the antibrain tumor efficacy of TMZ [245]. Another report shows the efficacy of CMV-specific T cell therapy, as it is reported that the expression of human cytomegalovirus (CMV) antigens in GBM tissues is pretty high. Distinct gene expression correlated with the better clinical response is recorded for the high grade brain tumor patients, who availed themselves of CMV-specific T cell therapy [246].

### 3.4. Effective Delivery of Therapeutic Peptides

Towards fulfilling the goal of effective therapy, recently selective peptides have been developed against brain cancer. Discovery of novel peptide as novel specific chemical entity is encouraged by the identification of several protein/peptide receptors and tumor-related peptides/proteins, those expressed in brain cancer cells. Small sized, less toxic peptides are advantageous over the monoclonal antibodies (mAbs) and large proteins that have large size and high toxicity have poor rate of BBB crossing. Other major advantages of peptides are their BBB penetrating ability in brain tumors, ease of synthesis and modification, and good biocompatibility [247]. Chlorotoxin is such a peptide which selectively binds to glioma cells [248]. Somatostatin analogues, which can be defined as peptide receptor radionuclide therapeutic agents, are the only approved cancer therapeutic peptides in the market [249] and there are reports of their binding to the cellular receptors in brain tumors* in vivo *[250]. Another new approach of brain tumor therapy is developing vaccines consisting of peptides derived from the protein sequence of brain tumor-associated or specific antigens [251]. Autologous DC vaccine against CD133 (a marker of GBM), survivin peptide vaccine, rindopepimut (also known as CDX-110) against EGFRVIII, and so forth are the examples of peptide vaccines for high grade brain tumors and these are now under clinical trials [252–254].

### 3.5. Molecular Trojan Horses (MTH)

Recently a new technique is used to ferry drug molecules across the BBB, which is called Molecular Trojan Horse (MTH) mediated drug delivery. Delivery of particular substances to the brain after attaching them to a protein, which can cross BBB, is the main focus of this type of delivery system. One of the recent progresses of MTH is “Trojan horse liposome” (THL) technology [255–257]. The application of this technology to transvascular nonviral gene therapy of brain represents a potential way out of the transvascular brain gene delivery problem. The THL is constructed with PEG-conjugated lipids which encapsulate plasmid DNA encoding proteins or shRNA/siRNA. Marked decrease in expression of EGFR protein in the tumor region was noticed after using THL mediated RNAi gene therapy. This resulted in a 90% increase in survival time of brain tumor patients [258].

### 3.6. Drug Delivery Targeting Brain Cancer Stem Cells

Cancer stem cells (CSCs) are the tumor initiating cells present in the tumor niche. These cells cause drug resistance, metastasis, and relapse of cancer. Most of the current chemotherapeutic molecules are able to destroy the cancer cells but not the CSCs. Thus, to kill these CSCs in brain tumors, effective treatment modalities are needed, which should also have the ability to cross the BBB; for example, curcumin encapsulated in nanoparticles caused a dose-dependent growth inhibition of brain tumor CSCs and neurospheres [259]. Other than this, targeting active genes like MGMT in brain CSCs by liposomes with anti-MGMT siRNA for oral Temozolomide therapy and destruction of brain CSCs niche by mAb-vectorized SWNT (single-walled carbon nanotubes) for hypothermic treatment also resulted in destruction of CSCs [260, 261]. The efficacy of the CSC targeting drugs can be improved by optimisation of chemo- and nanotherapies, novel gene-silencing techniques, and drug efflux inhibition techniques which may increase survivability of the brain tumor patients.

## 4. Concluding Remarks

Modern era of brain cancer therapy is characterized by novel target specific drugs with efficient delivery strategies. However, the prognosis and median survival of the brain tumor patients are not satisfactory till date. This is due to molecular heterogeneity of the brain tumors, presence of CSCs, and lack of effective drug delivery because of the presence of BBB. Rapid progress is needed in the sector of brain tumor characterization and BBB research. Till now, most effective drugs for brain tumor therapies are Temozolomide, Procarbazine, Carmustine (BCNU), Lomustine (CCNU), and Vincristine. Better modification of these drugs or identification of new chemical entities with enhanced efficacy and low side effect is always commendable. Alternatively, identification of drugs which can modulate BBB components or transporter systems could be an effective future strategy. Another potential future approach is combinatorial therapy, where through BBB destruction/modification, tumor cells/CSCs could be targeted easily. Modern techniques like nanotherapy may facilitate this kind of approach. Therefore, future research is needed to focus on the development of more specific targeting strategies to cure brain cancer, overcoming the above-mentioned difficulties arising due to the presence of the BBB.

## Figures and Tables

**Table 1:** Type of common brain cancers and their BBB status.

Type of brain tumors		Origin	Involvement of BBB	Status of BBB
Primary	Astrocytomas			
	Pilocytic astrocytoma (grade I)	Usually from astrocytes of cerebellum	Yes	Not well formed
	Fibrillary/mixed oligo astrocytoma (grade II)	From neoplastic astrocytes	Yes	Mostly intact
	Anaplastic astrocytoma (grade III)	From brain astrocytes which infiltrate through white matter of cerebral hemisphere, dura, and spinal fluid	Yes	Altered or disrupted
	Glioblastoma multiforme (GBM) (grade IV)	From glial cells	Yes	Altered or disrupted
	Oligodendrogliomas	From oligodendrocytes and glial precursor cells	Yes	Mostly intact
	Ependymomas	From ependyma	Yes	Intact
	Meningiomas	From meninges of brain and central nervous system	No	—
	Schwannomas	From Schwann cells	No	—
	Craniopharyngiomas	From pituitary gland embryonic tissue	Yes	Intact or disrupted
	Germinomas	Germ cell tumors from pineal gland	No	—
	Medulloblastomas	From cerebellum, below the tentorium of brain	Yes	Intact
	Pineocytoma	From pineal parenchyma	No	—
	Pineoblastoma	From pineal parenchyma	No	—

Secondary	Different metastatic cancers to brain	From cancers like breast, lung, bowel, kidney, ovary, and skin	Yes	Intact or disrupted

**Table 2:** Recent modifications of few important brain tumor drugs.

Drug name	Mode of action	Modification type	Examples	Usual route of administration	Targeted brain tumor type	Reference
Temozolomide	Alkylating agent	Nanoparticle based	Polysorbate-80 coated PBCA nanoparticles as feasible carrier for TMZ delivery to the brain			[193]
			Transferrin-appended PEGylated nanoparticles for TMZ delivery to brain			[194]
			TMZ solid lipid nanoparticles (TMZ-SLNs)	Oral	Glioblastoma multiforme	[195]
			Polysorbate-80 coated TMZ loaded PLGA based supermagnetic nanoparticles			[196]
			TMZ loaded in PLGA nanoparticle			[197]
			TMZ loaded in chitosan nanoparticle			[198]
			TMZ loaded in albumin nanoparticle			[199]

Carmustine (BCNU)	Alkylating agent	Liposomes, polymer microchips, and microspheres	Gliadel			[200]
		Nanoparticles	Chitosan surface-modified poly(lactide-co-glycolide) nanoparticles loaded with BCNU	Wafer implant/IV/oral	Glioblastoma multiforme, medulloblastoma, and low grade astrocytoma	[201]
			Catanionic solid lipid nanoparticles (CASLNs) carrying BCNU			[202]
			BCNU-loaded poly(lactic acid) (PLA) nanoparticle			[203]

Doxorubicin (DOX)	Anthracyclines, inhibiting nucleic acid synthesis	Liposome	Long-circulating PEGylated liposomes to cross blood brain barrier			[204]
		Nanoparticle	Cationic solid lipid nanoparticles (CASLNs), loaded with DOX	IV	Glioblastoma multiforme	[205]
			Human serum albumin nanoparticles loaded with DOX			[206]

Lomustine (CCNU)	Alkylating nitrosourea compound	Liposomes or microcapsules	Administration of CCNU-Lips and inclusion complex solution of CCNU with hydroxypropyl-*β*-cyclodextrin (CCNU-Sol)	Oral	Oligodendrogliomas and mixed oligoastrocytomas	[207]

Vincristine (Oncovin)	Vinca alkaloid	Liposome	Vincristine sulfate liposome, PEGylated liposome	IV	Anaplastic oligoastrocytoma and oligodendroglioma, metastatic secondary brain tumors	[208, 209]

Cisplatin	Platinum-containing anticancer drugs	Liposome	Transferrin-modified cisplatin liposome Cis-lipo(Tf)	IV	Glioma, medulloblastoma, and other types of brain tumors	[210]

Carboplatin	Platinum-based antineoplastic agents	Liposomes	Liposomal carboplatin	IV	Glioma, medulloblastoma, and other types of brain tumors	[211]

Methotrexate	Antimetabolite and antifolate	Nanoparticle	Magnetic nanoparticles	Oral/injection	Malignant brain tumors, brain lymphoma	[212]

Etoposide (ETP)	Topoisomerase inhibitor	Nanoparticle	ETP-encapsulated cationic solid lipid nanoparticles (ETP-CASLNs) grafted with 5-HT-moduline	IV/oral	Malignant brain tumors	[213]
			Liposomal etoposide			[211]

Actinomycin (dactinomycin)	Polypeptide antibiotics	Liposome	Liposome encapsulated actinomycin	IV	Secondary brain tumor, child brain tumor	[214]

Irinotecan	DNA topoisomerase I inhibitor	Liposome	Nanoliposomal irinotecan	IV	Glioblastoma multiforme	[215]

Paclitaxel (Taxol)	Taxanes	Chemical	Tx-67,10-O-deacetylpaclitaxel 10-monosuccinyl ester			[216]
		Liposomes	Polysorbate 80 coated poly (*ɛ*-caprolactone)-poly (ethylene glycol)-poly (*ɛ*-caprolactone) (PCEC) micelles	IV	High grade glioma, oligodendroglioma	[217]
			Paclitaxel plus artemether liposomes			[218]

## Abbreviations

BBB: Blood brain barrier
GBM: Glioblastoma multiforme
CSF: Cerebrospinal fluid
VE: Vascular endothelium
BMEC: Brain macrovascular endothelial cells
CNS: Central nervous system
ZO: Zonula occludens
JAM: Junction adhesion molecules
GLUT1: Glucose transporter 1
ATP: Adenosine triphosphate
ABC: ATP-binding cassette
SLC: Solute carrier
P-gp: P-glycoprotein
MDR: Multidrug resistant
BCRP: Breast cancer resistance protein
MRP: Multidrug resistance proteins
TMD: Transmembrane domains
POT: Proton coupled oligopeptide transporters
PEPT: Peptide transporters
PHT: Peptide/histidine transporter
PTS: Peptide transport system
MCT: Monocarboxylate transporters
TAT: T-type amino acid transporter
OATP: Organic anion transporters polypeptides
OAT: Organic anion transporters
OCT: Organic cation transporters
OCTN: Organic cation transporter novel
ENT: Equilibrative nucleoside transporters
CNT: Concentrative nucleoside transporters
VEGF: Vascular endothelial growth factor
CED: Convection enhanced diffusion
LDL: Low density lipoprotein
RMP: Receptor-mediated permeabilizer
MRI: Magnetic resonance imaging
RES: Reticuloendothelial system
PEG: Polyethylene glycol
GFAP: Glial fibrillary acidic proteins
NP: Nanoparticle
EGFR: Epidermal growth factor receptor
EGFRvIII: Epidermal growth factor receptor variant III
SLN: Solid lipid nanoparticle
HSV: Herpes simplex virus
MGMT: O6-Methylguanine-DNA methyltransferase
CMV: Cytomegalovirus
mAbs: Monoclonal antibodies
DC: Dendritic cell
MTH: Molecular Trojan Horse
THL: Trojan horse liposome
shRNA: Short hairpin RNA
siRNA: Small interfering RNA
RNAi: RNA interference
CSC: Cancer stem cell
SWNT: Single-walled carbon nanotubes.
